# SPIM workflow manager for HPC

**DOI:** 10.1093/bioinformatics/btz140

**Published:** 2019-02-25

**Authors:** Jan Kožusznik, Petr Bainar, Jana Klímová, Michal Krumnikl, Pavel Moravec, Václav Svatoň, Pavel Tomančák

**Affiliations:** 1 Department of Computer Science, FEECS VSB–Technical University of Ostrava, Ostrava, Czech Republic; 2 IT4Innovations, VSB–Technical University of Ostrava, Ostrava, Czech Republic; 3 Max Planck Institute of Molecular Cell Biology and Genetics, Dresden, Germany

## Abstract

**Summary:**

Here we introduce a Fiji plugin utilizing the HPC-as-a-Service concept, significantly mitigating the challenges life scientists face when delegating complex data-intensive processing workflows to HPC clusters. We demonstrate on a common Selective Plane Illumination Microscopy image processing task that execution of a Fiji workflow on a remote supercomputer leads to improved turnaround time despite the data transfer overhead. The plugin allows the end users to conveniently transfer image data to remote HPC resources, manage pipeline jobs and visualize processed results directly from the Fiji graphical user interface.

**Availability and implementation:**

The code is distributed free and open source under the MIT license. Source code: https://github.com/fiji-hpc/hpc-workflow-manager/, documentation: https://imagej.net/SPIM_Workflow_Manager_For_HPC.

**Supplementary information:**

Supplementary data are available at *Bioinformatics* online.

## 1 Introduction

Modern microscopes generate vast amounts of image data that require complex processing. For example, individual acquisitions in Selective Plane Illumination Microscopy (SPIM) can produce several terabytes of images due to very high spatiotemporal resolution. Consequently, processing a typical SPIM dataset on a single computer in a timely manner is often not possible, and employment of high-performance computing (HPC) resources is essential.

Approaches involving HPC clusters often require direct login access to the cluster as well as some expertise in command line operation. Since these two pre-requisites may be unavailable to many researchers, deployment of data processing to remote HPC clusters directly from the graphical user interface of a broadly used image analysis platform would substantially lower the entry barrier to this type of parallel processing.

Here we introduce such a solution as a plugin for Fiji (“Fiji Is Just ImageJ”), an open-source platform for biological image analysis (Rueden *et al.*, 2017; Schindelin *et al.*, 2012). As an application example for the proposed Fiji parallel processing framework we use a complex multi-step processing workflow for large SPIM datasets (Schmied *et al.*, 2016).

## 2 Materials and methods

Accessing a remote HPC cluster is often burdened by administrative overhead due to more or less complex security policies enforced by HPC centers. This barrier can be substantially lowered by employing a middleware tool based on the HPC-as-a-Service concept (Parashar *et al.*, 2013). To facilitate access to HPC from the Fiji environment, we utilize a HEAppE Middleware framework allowing end users to access an HPC system through web services and remotely execute pre-defined tasks. HEAppE is designed to be universal and applicable to various HPC architectures (see Section 1.2 of the Supplementary Material).

We developed a Fiji plugin relying on HEAppE, which enables users to control workflows running on remote HPC resources. As a representative workflow we use a *Snakemake*-based SPIM data processing pipeline operating on large multi-view SPIM image datasets (Schmied *et al.*, 2016), see Section 1.1 of the Supplementary Material. The *Snakemake* workflow engine resolves dependencies between subsequent steps and executes in parallel any tasks appearing to be independent, such as processing of individual time points of a time-lapse acquisition.

**Fig. 1.**
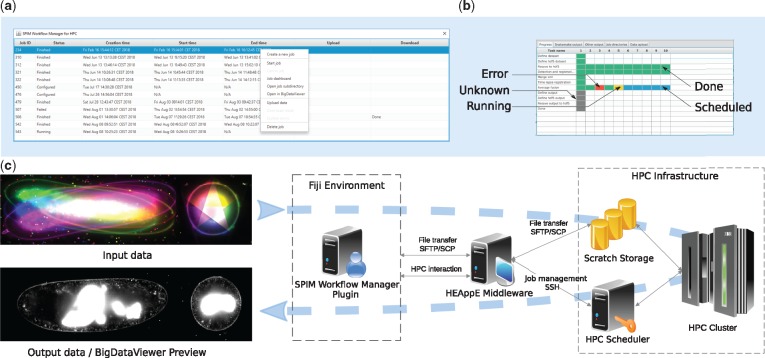
An overview of SPIM data processing on an HPC cluster: (**a**) a screenshot of the SPIM Workflow Manager plugin main window with 11 jobs and a context menu; (**b**) the dashboard for a selected job processing 10 time points of a SPIM recording; (**c**) an overview of the proposed solution—left: unregistered and fused SPIM data, middle: the SPIM data processing workflow in Fiji relying on the HEAppE Middleware framework, right: an example of an HPC infrastructure

Upon plugin invocation from the application menu, the user is prompted for HEAppE credentials. Following a successful login, the main window containing all jobs arranged in a table is displayed (Fig. 1a). In this context, the term *job* is used for a single pipeline run with specified parameters. The plugin actively queries information on the created jobs from HEAppE and updates the table as appropriate.

For creating a new job, the plugin provides a wizard allowing the user to specify input and output data paths, as well as to set up a configuration file *config.yaml*, which characterizes the dataset and defines settings for individual tasks. The plugin supports uploading of local input image data to the remote HPC resource, providing information on the progress and estimated remaining time as well as resumption of interrupted transfers.

Once a job execution is selected by the user, the configuration file is sent to the cluster via HEAppE. The user can examine a detailed progress dashboard showing current states of all individual computational tasks for the selected job (Fig. 1b) as well as output logs useful for debugging.

Following a successfully finished pipeline, the user can interactively examine the processed SPIM image data using the BigDataServer (Pietzsch *et al.*, 2015) as well as download resultant data and a summary file containing key information about the performed job. Importantly, the user can edit the corresponding local configuration file in a common text editor, and restart an interrupted, finished or failed job. For further details on the developed plugin, see Section 1.3 of the Supplementary Material.

## 3 Results

The test dataset we used was a 90 time-point SPIM acquisition of a *Drosophila melanogaster* embryo (see Section 1.4.1 of the Supplementary Material). The dataset consisted of 170 GB of image data.

Using the developed plugin, we transferred the dataset from MPI-CBG in Dresden, Germany to IT4Innovations in Ostrava, Czech Republic and executed the pipeline there on the Salomon supercomputer (see Section 1.4.2 of the Supplementary Material). Following successful processing, the resultant data were downloaded back to the computer in Dresden.

The data transfer and pipeline execution on Salomon using 90 nodes took 9 h 37 min. For comparison, processing of the same dataset on a common workstation took 23 h 56 min (for details see Section 1.4.3 of the Supplementary Material). The results show that despite the data transfer overhead, a significant speedup of SPIM image analysis has been achieved by employing HPC resources.

## 4 Conclusions

The developed plugin enables researchers to run computationally intensive tasks on a remote HPC cluster directly from the Fiji environment, significantly enhancing the user experience.

Transferring voluminous data to a remote HPC cluster can be time-consuming, however the imposed overhead is compensated for with higher processing power offered by the cluster.

Our cluster-mediated data processing approach is applicable to analogous workflows and HPC infrastructures supporting the HPC-as-a-Service concept.

## Funding

This work was supported by the European Regional Development Fund in the IT4Innovations National Supercomputing Center—path to exascale project [project number CZ.02.1.01/0.0/0.0/16_013/0001791] within the Operational Programme Research, Development and Education. 

This work was also supported by the Ministry of Education, Youth and Sports from the Large Infrastructures for Research, Experimental Development and Innovations project “IT4Innovations National Supercomputing Center – LM2015070”.

*Conflict of Interest*: none declared.

## Supplementary Material

btz140_Supplementary_Data
