# Supplementary material for: SPIM workflow manager for HPC
Source: Bioinformatics. 2019 Feb 25;35(19):3875–6. doi: 10.1093/bioinformatics/btz140 (PMC6761944; doi:10.1093/bioinformatics/btz140)
Supplement: btz140_Supplementary_Data [file btz140_supplementary_data.zip › btz140_suppl_data/Bioinformatics___supplementary_data.pdf]

SPIM Workflow Manager for HPC

Jan Kožusznik[1,2], Petr Bainar[2], Jana Klímová[2], Michal Krumnikl[1,2], Pavel Moravec[1,2], Václav Svatoň[2], and Pavel Tomančák[2,3]

[1]Department of Computer Science, FEECS VŠB – Technical University of Ostrava, Ostrava-Poruba, Czech Republic
[2]IT4Innovations, VŠB – Technical University of Ostrava, Ostrava-Poruba, Czech Republic
[3]Max Planck Institute of Molecular Cell Biology and Genetics, Dresden, Germany

1 Supplementary Data

This document contains supplementary data to the original paper *SPIM Workflow Manager for HPC*.

1.1 SPIM Data Processing Pipeline

Selective Plane Illumination Microscopy (SPIM) typically images living biological samples from multiple angles (views) collecting several 3D image stacks to cover the entire biological specimen. The 3D image stacks, representing one time point in a long-term time-lapse acquisition, need to be registered to each other, which is typically achieved using fluorescent beads as fiduciary markers [Preibisch et al., 2010]. After the registration, the individual views within one time point need to be combined into a single output image either by content-based fusion [Preibisch et al., 2008] or multi-view deconvolution [Preibisch et al., 2014]. The living specimen can move during acquisition, necessitating an intermediate step of time-lapse registration [Preibisch et al., 2010]. Whereas processing of individual time points is embarrassingly parallel, the time-lapse registration can be performed on a single compute node without the need for parallelization.

The sheer amount of SPIM data requires conversion from raw microscopy data to Hierarchical Data Format (HDF5) for efficient input/output access and visualization in Fiji's BigDataViewer (BDV) [Pietzsch et al., 2015]. BDV uses an XML file to store experiment metadata (i.e. number of angles, time points, channels etc.). Although the conversion to HDF5 is a parallelizable procedure, further updating the XML file downstream in the pipeline is not; and per-time point XML files have to be created and then merged after completion of the registration and fusion steps. Consequently, the parallel processing of individual

time points on an HPC resource (conversion to HDF5, registration, fusion and deconvolution) is interrupted by non-parallelizable steps (time-lapse registration and XML merging).

In the parallel processing workflow relying on the *Snakemake* engine [Schmied et al., 2016], pipeline input parameters are entered by a user into a *config.yaml* configuration file. In the first step, the *.czi* raw data are concurrently resaved into the HDF5 container on the cluster. Similarly, the individual time points are registered in parallel using fluorescent beads as fiduciary markers on the cluster. Subsequently, a non-parallel job executed by *Snakemake* consolidates the registration XML files into a single one, followed by time-lapse registration using the beads segmented during the spatial registration step. After this, the pipeline diverges into either parallel content-based fusion or parallel multi-view deconvolution. To achieve this divergence in practice, the *Snakemake* pipeline is launched from the Fiji plugin using *config.yaml* files set to execute content-based fusion and deconvolution respectively. In the final stage of the pipeline, the fusion/deconvolution output is saved into a new HDF5 container.

1.2 HEAppE Middleware

HPC-as-a-Service is a well-known term in the area of high performance computing. It enables users to access an HPC infrastructure without the need to buy and manage their own physical servers or data center infrastructure. This approach further lowers the entry barrier for users who are interested in utilizing massive parallel computers but do not have the necessary level of expertise in this area.

To provide this simple and intuitive access to the supercomputing infrastructure, an application framework called High-End Application Execution (HEAppE) Middleware[1] has been developed. This middleware provides HPC capabilities to the users and third-party applications without the need to manage the running jobs from the command-line interface of the HPC scheduler on the cluster.

HEAppE also provides the mapping between the external users and internal cluster service accounts that are being used for the actual job submission to the cluster. It simplifies access to the computation resources from the security and administrative points of view. For security purposes, users are permitted to run only a pre-prepared set of so-called command templates. Each command template defines an arbitrary script or an executable file which is to be run on the cluster, a set of input parameters modifiable at runtime, any dependencies or third-party software it might require, and the type of queue that should be used for the processing.

HEAppE has already been successfully used in several projects; for example, providing What-If analysis in the crisis decision support system Floreon+ [Svatoň et al., 2018], satellite image data analysis, and in the area of molecular diagnostics and personalized medicine.

[1] http://heappe.eu

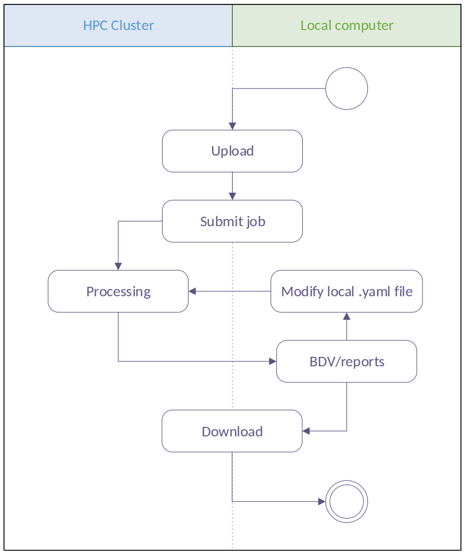

Figure 1: Data processing workflow with the developed plugin

1.3 SPIM Workflow Manager for HPC plugin

To provide users with an intuitive interface for managing pipeline jobs[2], we created a Fiji plugin[3] accessible directly from the application menu. This plugin communicates with HEAppE, which submits requests for pipeline job executions to the cluster where the *Snakemake* engine is launched.

Upon plugin invocation, the user needs to be authenticated by verifying HEAppE credentials[4]. Additionally, a local working directory must be defined. Later on it will contain specific job subdirectories, each of them serving as a staging area for a *config.yaml* configuration file specifying pipeline parameters.

Following a successful login, the main window providing top-level information on all jobs is displayed. Figure 1 displays the typical workflow described in the following sections.

The plugin comprises a job configuration wizard where the user can define input and output paths as well as opt for using a pre-defined *config.yaml* configuration file. This file can be edited locally in a common text editor, allowing the user to swiftly modify parameters for existing jobs. Once the job configuration is confirmed by the user, the plugin obtains a unique job ID from HEAppE, and automatically creates a job subdirectory named identically to the job ID in the working directory.

In the next step, the user typically uploads input image data. The main window constantly updates the user about the upload progress, conveniently

[2]In this context, the term *job* is used for a single SPIM data processing pipeline run with specified parameters

[3]The plugin can be downloaded from http://sites.imagej.net/P2E-IT4Innovations/

[4]Credentials required for authentication can be applied for by contacting the authors

displaying the estimated remaining time. Importantly, the user may pause the data upload and resume it later on from the context menu in the main window. This mechanism takes care of any unexpected interruptions of the connectivity during lengthy data transfer.

Once files are transferred, the user starts the job execution. The job metadata together with the *config.yaml* configuration file are sent to the cluster via HEAppE, which is responsible for the job life cycle from this point on. The *Snakemake* engine ensures that consecutive steps identified as independent are executed in parallel as separate tasks on computational nodes where Fiji instances are run in headless mode.

The main window providing an overview of all jobs periodically retrieves job states from HEAppE and updates the table.

In addition, the user can display a detailed progress dashboard showing the contemporaneous states of all individual computational tasks for the selected job. The dashboard also includes panes for displaying the *Snakemake* output, thereby providing useful debugging information.

Once the pipeline has successfully finished, the user can interactively examine the processed SPIM image data residing on the remote cluster file system using the BigDataServer [Pietzsch et al., 2015] as well as download resultant data and a summary file containing key information about the performed job.

The user can edit the corresponding local configuration file in a common text editor, and restart an interrupted, finished, or failed job. In this way, interactive debugging on the remote cluster is enabled.

1.4 Plugin Benchmarking

Execution of the *Snakemake* pipeline from the implemented Fiji plugin was tested on the Salomon supercomputer at IT4Innovations in Ostrava, Czech Republic and compared with a measurement run on a local workstation at Max Planck Institute of Molecular Cell Biology and Genetics (MPI-CBG) in Dresden, Germany [Schmied et al., 2016].

The measurement was comprised of the total time taken for data upload from MPI-CBG to IT4Innovations, pipeline execution on the Salomon supercomputer and downloading the resultant data back to the workstation in Dresden.

1.4.1 Dataset Description

The test dataset we used was a 90 time-point SPIM acquisition of a *Drosophila melanogaster* embryo expressing FlyFos fluorescent GFP fusion reporter for the *nrv2* gene [Sarov et al., 2016]. The embryo was imaged with a Lightsheet Z.1 SPIM microscope (Carl Zeiss Microscopy) from 5 views every 15 minutes from the cellular blastoderm stage until the late stages of fruitfly embryogenesis. The same dataset was previously used to benchmark the performance of the Snakemake SPIM processing pipeline on a local cluster [Schmied et al., 2016].

Figure 2 shows results of registration and fusion.

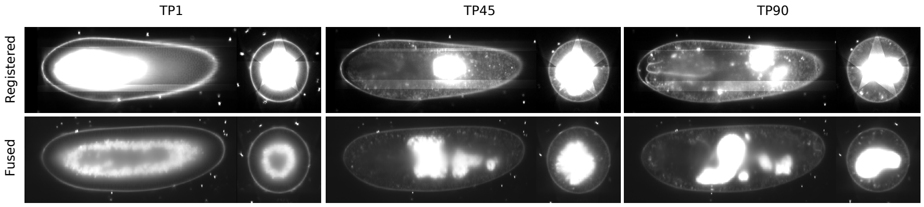

Figure 2: Visualization of SPIM data at different stages of processing. Lateral (left) and cross-sectional (right) views of registered and fused embryo volumes at time points 1, 45 and 90.

1.4.2 Technical Specification of Used Resources

Salomon supercomputer consists of 1 008 compute nodes, each of which is equipped with 2×12-core Intel Xeon E5-2680v3 (2.5 GHz CPU; average CPU PassMark 18 626) processors and 128 GB RAM, providing a total of 24 192 compute cores using x86-64 architecture and 129 TB RAM. The total theoretical peak performance reaches 2 000 TFLOPS.

The workstation at MPI-CBG has the following specification: 2×6-core Intel Xeon E5-2630 (2.3 GHz CPU); 128 GB RAM; 2× NVIDIA Quadro 4000 (256 CUDA cores); 4× 4 TB SATA disks in RAID 5 configuration.

1.4.3 Results

We carried out 9 measurements in total. Table 1 shows the average duration of each of the individual experimental steps together with their standard deviations. Note that the resultant data size is approximately 215 GB, whereas the original input dataset volume had a size of 170 GB.

Table 1: Duration of individual experiment steps

	Local workstation	Salomon
Data upload	-	01:40:14 (± 00:11:13)
Processing	23:56:00	01:41:01 (± 00:27:28)
Data download	-	06:01:12 (± 00:17:51)
Total time	23:56:00	09:22:27 (± 00:40:55)

References

T. Pietzsch, S. Saalfeld, S. Preibisch, and P. Tomancak. Bigdataviewer: visualization and processing for large image data sets. *Nature methods*, 12(6):481, 2015.

S. Preibisch, T. Rohlfing, M. P. Hasak, and P. Tomancak. Mosaicing of single plane illumination microscopy images using groupwise registration and fast content-based image fusion. In *Medical Imaging 2008: Image Processing*, volume 6914, page 69140E. International Society for Optics and Photonics, 2008.

S. Preibisch, S. Saalfeld, J. Schindelin, and P. Tomancak. Software for bead-based registration of selective plane illumination microscopy data. *Nature methods*, 7(6):418, 2010.

S. Preibisch, F. Amat, E. Stamataki, M. Sarov, R. H. Singer, E. Myers, and P. Tomancak. Efficient bayesian-based multiview deconvolution. *Nature methods*, 11(6):645, 2014.

M. Sarov, C. Barz, H. Jambor, M. Y. Hein, C. Schmied, D. Suchold, B. Stender, S. Janosch, V. V. KJ, R. Krishnan, A. Krishnamoorthy, I. R. Ferreira, R. K. Ejsmont, K. Finkl, S. Hasse, P. Kämpfer, N. Plewka, E. Vinis, S. Schloissnig, E. Knust, V. Hartenstein, M. Mann, M. Ramaswami, K. VijayRaghavan, P. Tomancak, and F. Schnorrer. A genome-wide resource for the analysis of protein localisation in Drosophila. *eLife*, 5, 2016. ISSN 2050-084X. doi: 10.7554/eLife.12068.

C. Schmied, P. Steinbach, T. Pietzsch, S. Preibisch, and P. Tomancak. An automated workflow for parallel processing of large multiview spim recordings. *Bioinformatics*, 32(7):1112–1114, 2016. doi: 10.1093/bioinformatics/btv706.

V. Svatoň, M. Podhoranyi, R. Vavřík, P. Veteška, D. Szturcová, D. Vojtek, J. Martinovič, and V. Vondrák. Floreon+: A web-based platform for flood prediction, hydrologic modelling and dynamic data analysis. In *Dynamics in GIscience*, pages 409–422, 2018. ISBN 978-3-319-61297-3.
