# Supplementary material for: SPIM workflow manager for HPC
Source: Bioinformatics. 2019 Feb 25;35(19):3875–6. doi: 10.1093/bioinformatics/btz140 (PMC6761944; doi:10.1093/bioinformatics/btz140)
Supplement: btz140_Supplementary_Data [file btz140_supplementary_data.zip › btz140_suppl_data/Bioinformatics-supplementary_data.zip!/images/droso-crop.pdf]

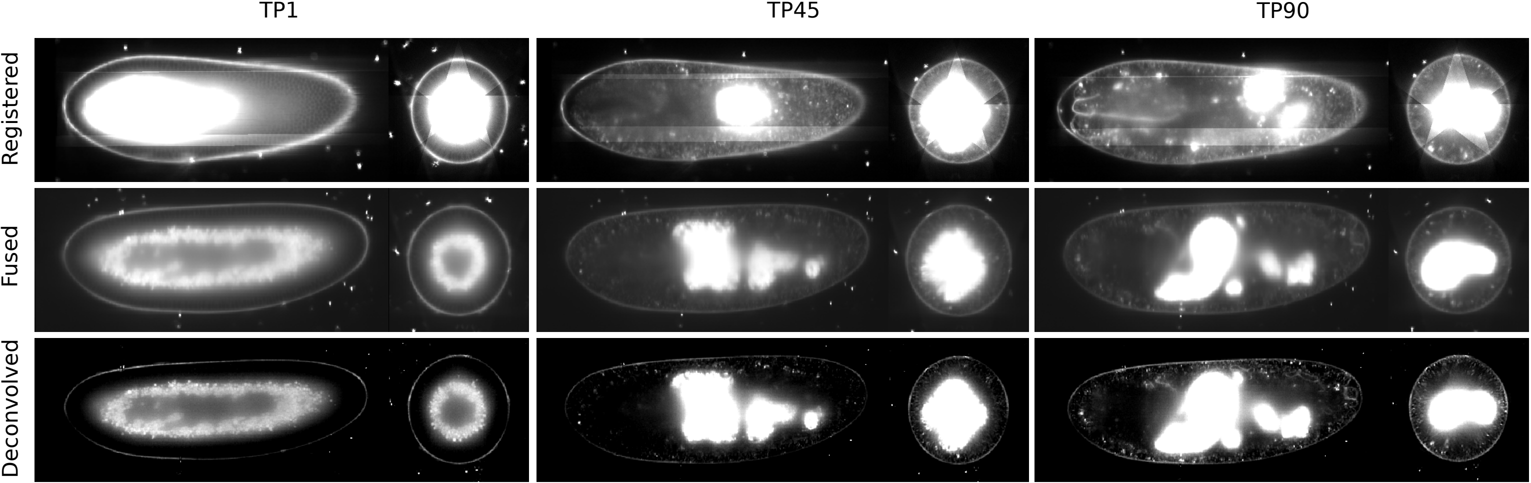

TP1
TP45
TP90
Registered
Fused
Deconvolved
