# Supplementary material for: SPIM workflow manager for HPC
Source: Bioinformatics. 2019 Feb 25;35(19):3875–6. doi: 10.1093/bioinformatics/btz140 (PMC6761944; doi:10.1093/bioinformatics/btz140)
Supplement: btz140_Supplementary_Data [file btz140_supplementary_data.zip › btz140_suppl_data/Bioinformatics-supplementary_data.zip!/images/screenshots-two-crop.pdf]

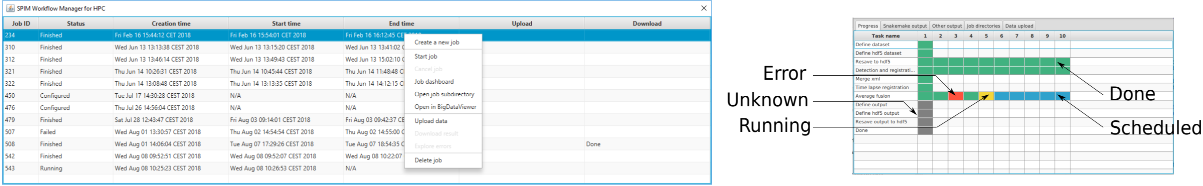
SPIM Workflow Manager for HPC
Job ID
Status
Creation time
Start time
End time
Upload
Download
234 Finished Fri Feb 16 15:44:12 CET 2018 Fri Feb 16 15:54:01 CET 2018 Fri Feb 16 16:12:45 CET
310 Finished Wed Jun 13 13:13:38 CEST 2018 Wed Jun 13 13:15:20 CEST 2018 Wed Jun 13 13:41:02
312 Finished Wed Jun 13 13:46:14 CEST 2018 Wed Jun 13 13:49:43 CEST 2018 Wed Jun 13 15:02:10
321 Finished Thu Jun 14 10:26:31 CEST 2018 Thu Jun 14 10:45:44 CEST 2018 Thu Jun 14 11:48:48
322 Finished Thu Jun 14 13:08:48 CEST 2018 Thu Jun 14 13:13:35 CEST 2018 Thu Jun 14 14:12:15
450 Configured Tue Jul 17 14:30:28 CEST 2018 N/A N/A
476 Configured Thu Jul 26 14:56:04 CEST 2018 N/A N/A
479 Finished Sat Jul 28 12:43:47 CEST 2018 Fri Aug 03 09:14:01 CEST 2018 Fri Aug 03 09:42:37
507 Failed Wed Aug 01 13:30:57 CEST 2018 Thu Aug 02 14:54:54 CEST 2018 Thu Aug 02 14:55:00
508 Finished Wed Aug 01 14:06:04 CEST 2018 Tue Aug 07 17:29:26 CEST 2018 Tue Aug 07 18:54:35 Done
542 Finished Wed Aug 08 09:52:51 CEST 2018 Wed Aug 08 09:52:07 CEST 2018 Wed Aug 08 10:22:07
543 Running Wed Aug 08 10:25:23 CEST 2018 Wed Aug 08 10:26:53 CEST 2018 N/A
Create a new job
Start job
Cancel job
Job dashboard
Open job subdirectory
Open in BigDataViewer
Upload data
Download result
Explore errors
Delete job
Progress
Snakemake output
Other output
Job directories
Data upload
Task name
1 2 3 4 5 6 7 8 9 10
Define dataset
Define hdf5 dataset
Resave to hdf5
Detection and registrati...
Merge xml
Time lapse registration
Average fusion
Define output
Define hdf5 output
Resave output to hdf5
Done
Error
Unknown
Running
Done
Scheduled

(a)

(b)
